# Supplementary material for: PIEZO1 Promotes the Migration of Endothelial Cells via Enhancing CXCR4 Expression under Simulated Microgravity
Source: Int J Mol Sci. 2024 Jul 1;25(13):7254. doi: 10.3390/ijms25137254 (PMC11242226; doi:10.3390/ijms25137254)
Supplement: Supplementary file 1 [file ijms-25-07254-s001.zip › ijms-3064879-Supplemental information .pdf]

Supplemental information

PIEZO1 promotes the migration of endothelial cells via enhancing CXCR4 expression under simulated microgravity

Yuan Wang [1, #], Chengfei Li [1, #], Ruonan Wang[1], Xingcheng Zhao [1], Yikai Pan[1], Qian Zhang[1], Shuhan Li [1], Jieyi Fan[1], Yongchun Wang[1], Xiqing Sun[1, *]

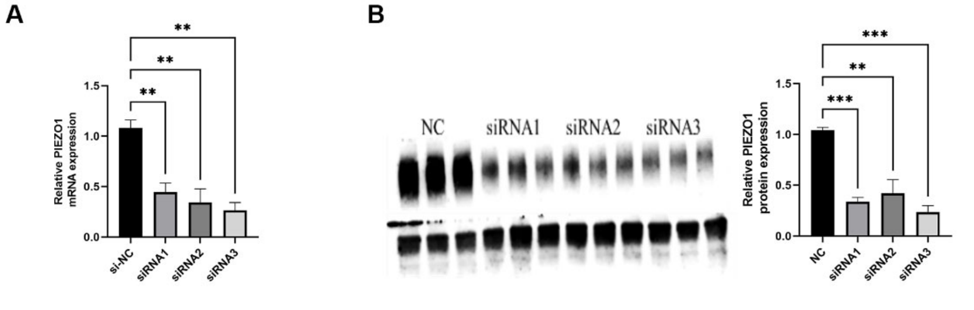

FIGURE S1. Analysis of transfection efficiency

A The mRNA expression levels of Piezo1 with Piezo1 siRNA1, 2, and 3 were examined by qRT-PCR in HUVECs.

B Western blot analysis, showing the protein level of Piezo1 with Piezo1 siRNA1, 2, and 3 in HUVECs. (The data represent the mean ± *SEM*, n = 3, $^{**}P < 0.01$, $^{***}P < 0.001$)

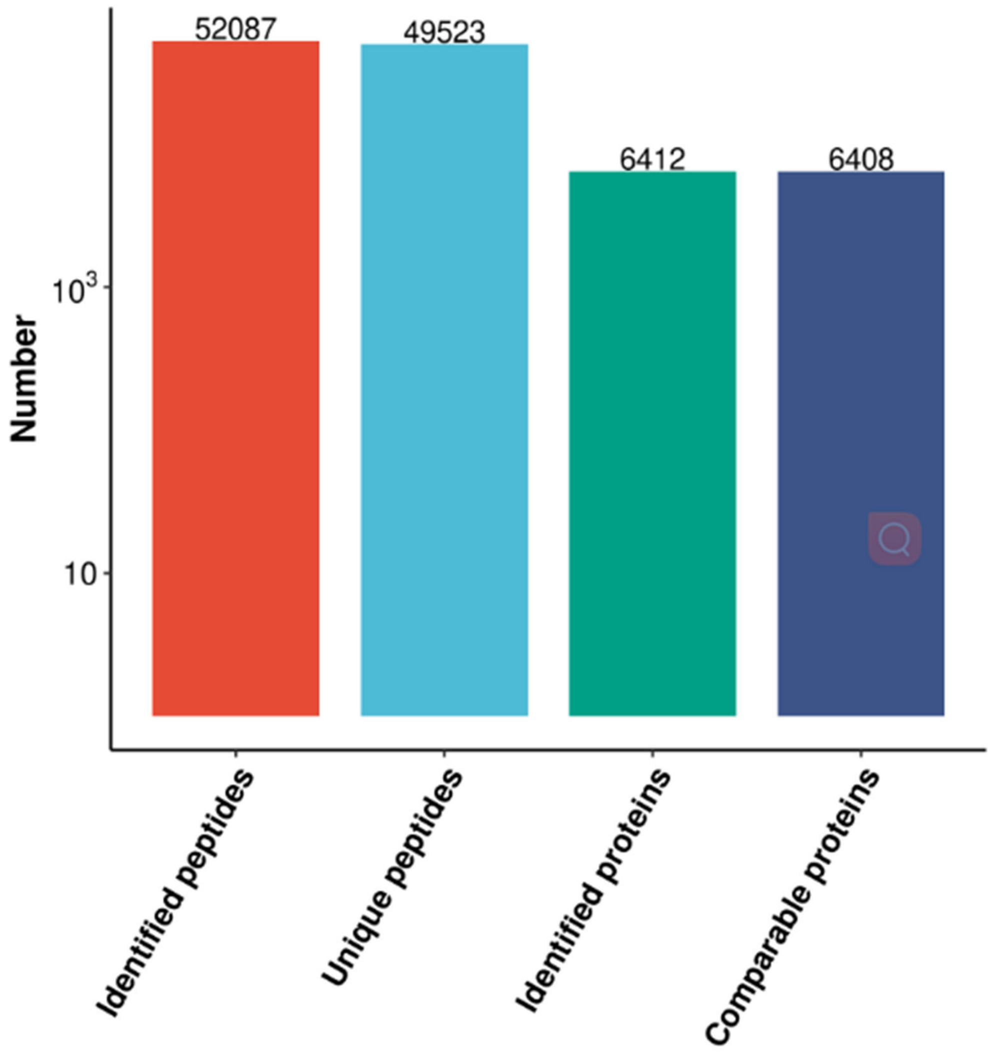

FIGURE S2 The results of LC-MS/MS

A total of 52087 peptides were identified with high confidence (false discovery rate < 1%), of which 49523 peptides were identified as unique peptides. Of these, 6412 proteins were identified, of which 6408 proteins were comparable proteins

	MG+siNC/CON+si-NC Ratio	MG+siNC/CON+si-C *P* value	MG+si-PIEZO1/MG+NC Ratio	MG+siPIEZO1/MG+si-NC *P* value
CXCR4	5.12	0.006	0.48	0.003

FIGURE S3 The expression of CXCR4 in proteomic analysis

Compared to Con+si-NC group, the expression of CXCR4 in MG+si-NC group was upregulated 5.12-fold. However, PIEZO1-knock down downregulated CXCR4 protein level 0.48-fold, compared to that in MG+si-NC group.
